# Ketone Bodies and Exercise Performance: The Next Magic Bullet or Merely Hype?

**DOI:** 10.1007/s40279-016-0577-y

**Published:** 2016-07-18

**Authors:** Philippe J. M. Pinckaers, Tyler A. Churchward-Venne, David Bailey, Luc J. C. van Loon

**Affiliations:** 1 NUTRIM, School of Nutrition and Translational Research in Metabolism, Maastricht University Medical Centre +, P.O. Box 616, 6200 MD Maastricht, The Netherlands; 2 BMC Racing Team, Eke, Belgium

## Abstract

Elite athletes and coaches are in a constant search for training methods and nutritional strategies to support training and recovery efforts that may ultimately maximize athletes’ performance. Recently, there has been a re-emerging interest in the role of ketone bodies in exercise metabolism, with considerable media speculation about ketone body supplements being routinely used by professional cyclists. Ketone bodies can serve as an important energy substrate under certain conditions, such as starvation, and can modulate carbohydrate and lipid metabolism. Dietary strategies to increase endogenous ketone body availability (i.e., a ketogenic diet) require a diet high in lipids and low in carbohydrates for ~4 days to induce nutritional ketosis. However, a high fat, low carbohydrate ketogenic diet may impair exercise performance via reducing the capacity to utilize carbohydrate, which forms a key fuel source for skeletal muscle during intense endurance-type exercise. Recently, ketone body supplements (ketone salts and esters) have emerged and may be used to rapidly increase ketone body availability, without the need to first adapt to a ketogenic diet. However, the extent to which ketone bodies regulate skeletal muscle bioenergetics and substrate metabolism during prolonged endurance-type exercise of varying intensity and duration remains unknown. Therefore, at present there are no data available to suggest that ingestion of ketone bodies during exercise improves athletes’ performance under conditions where evidence-based nutritional strategies are applied appropriately.

## Key Points

**Table:** 

There is increasing interest in ketogenic diets and the use of ketone body supplements within the athletic community.
Ketone bodies represent an alternative energy substrate, and may alter substrate metabolism under certain conditions such as starvation and after supplementation.
Our current understanding of ketone body kinetics during exercise is insufficient to warrant their use as an ergogenic aid in any practical sports setting.

## Introduction

Nutrients consumed before and during exercise training can support optimal performance by delaying the onset of fatigue and assisting in the recovery process by replenishing endogenous substrates stores. Particularly for endurance-type sports characterized by high rates, and absolute levels of energy expenditure, appropriate nutrient intake is essential to fuel exercise, delay the deterioration in performance, and promote post-exercise recovery [1–5]. For endurance-type sports, research has focused on improving carbohydrate availability to better meet the anticipated fuel demands of the competition [6]. However, alternative fueling strategies based on adaptation to a high-fat carbohydrate-restricted diet increase fat oxidation during exercise and, as such, may help to spare the body’s limited glycogen stores [7]. Although the focus of fat-based fueling strategies has been to enhance the capacity for fat oxidation during exercise, extreme carbohydrate restriction (e.g., <50 g/day) [8] also increases the production of ketone bodies, which may provide an additional energy substrate for the brain and skeletal muscle tissue [9, 10]. Recently, the ingestion of ketone body supplements has emerged as an alternative strategy to induce hyperketonemia [11], with considerable media speculation about ketone body supplements being used by professional cyclists [12]. This article will discuss what is currently known regarding ketone bodies within the context of exercise metabolism and performance, with an emphasis on the proposed use of ketone body supplements to improve exercise performance.

## What are Ketone Bodies?

Ketone bodies are lipid-derived organic compounds that can serve as a circulating energy source for tissues during starvation/fasting or prolonged exercise [13]. Under physiological conditions in which ample carbohydrate is available, or after an overnight fast, circulating ketone body concentrations are relatively low (~0.1–0.5 mmol/L) [14, 15]. However, under conditions of limited carbohydrate availability such as starvation (i.e., depletion of muscle and liver glycogen stores), fatty acid mobilization from adipose tissue is increased as a means to supply energy. Under these circumstances, some of the acetyl-CoA derived from fatty acids is converted to ketone bodies via hepatic mitochondria (up to ~150 g/day) [16, 17]. The ketone body acetoacetate (AcAc) can subsequently be enzymatically converted to β-hydroxybutyrate (β-OHB) or spontaneously degraded to acetone which is less abundant [13]. For clarification, although the term “ketone bodies” refers to the compounds AcAc, β-OHB, and acetone which are derived from acetyl-CoA, only AcAc and acetone are actual “ketones” containing a carbonyl group with two hydrocarbon atoms. β-OHB is a ketone body, but is technically not a ketone since one of its hydrocarbon atoms is replaced by a hydroxyl group [18]. While the majority of acetone is secreted through urine and lost via expiration, AcAc and β-OHB are transported in the bloodstream to extrahepatic tissues with a high metabolic demand such as the brain, heart, and skeletal muscle. Ketone bodies cross the plasma and mitochondrial membranes by monocarboxylate transporters and are converted back to acetyl-CoA and used as an alternative source of energy by the tricarboxylic acid (TCA) cycle [13, 14, 19]. In addition to serving as an alternative energy substrate, ketone bodies play an important role in the regulation of skeletal muscle substrate utilization [14], cellular signaling and transcription [13, 20], and may have various therapeutic implications (for a review on that topic see Veech [16] and Hashim and VanItallie [21]).

## Availability of Ketone Bodies

### Hyperketonemia from Endogenous Origin

After an overnight fast endogenous ketone body production amounts to ~0.25 mmol/min (or ~35 g/24 h) resulting in relatively low circulating ketone body concentrations (~0.1–0.5 mmol/L) [22–24]. However, with prolonged fasting/starvation (~5 days), the rate of ketone body production reaches levels of ~1–2 mmol/min (or 140–280 g/24 h) [23, 24], corresponding to a plasma concentration of ~7–10 mmol/L. Beyond 5 days without food, plasma ketone body concentrations plateau and rarely exceed ~10 mmol/L [22]. This upper limit of ketonemia has been suggested to be the result of an inherent feedback mechanism by which ketone bodies inhibit their own production via exerting an insulinotropic [25–27] and anti-lipolytic effect [28] (for a review on this topic, see Balasse and Féry [22] and Balasse [29]). Outside of prolonged fasting/starvation a ketogenic diet can increase circulating ketone body concentrations to ~1–2 mmol/L after 2–4 days [30, 31]. Typical ketogenic diets are characterized by high fat (~80 % of daily energy intake), low carbohydrate (~20–50 g/day or ~5 % of daily energy intake), and moderate to relatively low protein intake (~15 % of daily energy intake) [9, 32]. However, the level of ketonemia induced via a ketogenic diet is largely dependent on the amount of carbohydrate ingested and can reach ~7–8 mmol/L following sustained periods of severe carbohydrate intake restriction (i.e., keto adaptation) [9].

Next to dietary interventions, prolonged physical exercise performed in the fasted state also stimulates ketogenesis during exercise [33–35] and results in post-exercise hyperketonemia [22, 34–38]. For example, ketone body concentrations can reach ~0.5–1.0 mmol/L in response to 2 h of exercise performed in an overnight fasted state [33] and can subsequently increase to ~1–4 mmol/L during early post-exercise recovery [22, 34–38]. The extent of exercise-induced hyperketonemia during and after exercise is influenced by the intensity and duration of the exercise performed as well as the nutritional status [22, 35, 38, 39]. For example, carbohydrate intake in close temporal proximity to exercise strongly attenuates the exercise induced increase in plasma ketone body concentrations [22, 35, 38–41]. Interestingly, well-trained individuals demonstrate an attenuated rise in plasma ketone body concentrations during and after exercise when compared to untrained individuals [34, 36, 37]. This has been attributed to a training mediated attenuation in the post-exercise increase in free fatty acid (FFA) concentrations [34, 36], and/or to increased activity of the enzymes involved in ketone body utilization [42, 43].

Evaluating ketone body kinetics in an overnight fasted state [44–46] or in response to prolonged fasting/starvation [33, 47–49] is of little practical relevance to endurance athletes seeking to apply optimal fueling strategies for competition. Furthermore, although ketogenic diets have been proposed to benefit various types of athletes [9, 10, 32], induction of nutritional ketosis via a ketogenic diet is dependent upon the depletion of hepatic and muscle glycogen reserves, thereby increasing circulating FFA and endogenous ketone body production [50]. However, a high fat, low carbohydrate ketogenic diet may reduce the capacity to utilize carbohydrate [51], thereby compromising exercise training intensity [8] and limiting exercise performance [52–54], particularly during moderate to high-intensity exercise activities, such as marathon running and hill climbs, sprints, and accelerations during a cycling race [55]. Therefore, orally ingested ketone body supplements may represent a more practical alternative to increase circulating ketone body concentrations in athletes [11, 15] since they do not require adherence to a high fat, low carbohydrate ketogenic diet to induce ketosis.

### Hyperketonemia from Exogenous Origin

Since the 1960s, studies examining the effects of exogenously provided ketone bodies have utilized ketone body salts administered either intravenously [47, 56–61] or orally [37]. Currently commercially available ketone body supplements (salts) provide ~8–12 g of β-OHB and ~1 g of sodium per serving, and serve as a means to rapidly increase circulating ketone body availability. Recently, ketone esters [i.e., (*R*)-3-hydroxybutyl (*R*)-3-hydroxybutyrate] have emerged as a more practical and applicable way to increase the availability of blood ketone bodies [15, 62–64]. Following ingestion, ketone esters [i.e., ketone monoester (*R*)-3-hydroxybutyl (*R*)-3-hydroxybutyrate] are cleaved in the gut and absorbed via the gut epithelium and monocarboxylate transporters into the circulation, or undergo first-pass metabolism in the liver to ketone bodies [11]. In comparison to starvation and/or a ketogenic diet which can take days to elicit a robust increase in ketone body concentrations, ingestion of ketone body supplements can rapidly increase plasma ketone body concentrations, reaching peak levels within 1–2 h [15, 62]. Plasma ketone body concentrations may increase to ~3 mmol/L 1 h after ingestion of ~400 mg of the ketone ester per kg body weight [31, 45, 46] or alternatively reach similar concentrations 10 min after ingesting ~600 mg/kg body weight, eventually resulting in plasma ketone body concentrations of ~6 mmol/L 45 min after ingestion [47].

Although ketone body supplements may induce a state of hyperketonemia, the ketone body concentrations achieved following ingestion appear to be influenced by concomitant food intake [41]. In this regard, recent data published in abstract form [41] reported that postprandial ketone body concentrations following ingestion of a ketone ester supplement (395 mg/kg body weight) were strongly influenced by baseline nutritional status. Specifically, an attenuated increase in peak plasma β-OHB concentrations (fed: 2.1 ± 0.2 mmol/L vs. fasted: 3.1 ± 0.1 mmol/L), and a 60 % reduction in β-OHB area under the curve was demonstrated when the ketone esters were ingested after consumption of a mixed meal when compared to consumption under fasted (post-absorptive) conditions [41]. These findings suggest that co-ingestion of ketone esters with other nutrients may impact gastric emptying and/or tissue uptake of the ingested ketone bodies. This finding may have implications regarding the effectiveness of ketone ester supplementation in athletes, who ingest large amounts of carbohydrate during a competitive event and adapt their nutrient intake during competition according to their specific nutritional needs [65]. Alternatively, whether ketone body supplementation impacts or compromises the intake of other important substrates, such as carbohydrate, may be an important consideration for endurance athletes who often struggle to ingest adequate amounts of carbohydrate during competition [66]. Finally, an additional consideration is how ketone body intake during exercise is tolerated by athletes. Normal healthy subjects, consuming 714 mg/kg of a milk-based ketone ester drink three times a day, were reported to experience feelings of discomfort, including abdominal distention and headaches [15]. However, it should be noted that the milk-based test drink volume amounted to 1.1 L per serving, making it difficult to differentiate between the effects of the ketone esters per se and simply the volume of the drinks that were ingested [15].

## Factors Influencing Ketone Body Utilization

Although ketone bodies may serve as an alternative oxidative fuel source, factors including tissue type (i.e., skeletal muscle vs. brain) [22, 58], exercise/training status [34, 36–38], circulating ketone body concentrations [33, 58], and skeletal muscle fiber type (i.e., type I vs. type II muscle fibers) [42, 43] have been shown to influence ketone body metabolism. For example, reduced ketone body uptake and oxidation rates were observed by skeletal muscle when compared to the brain when assessed under resting conditions [58]. Specifically, although ketone body uptake and oxidation by the brain appears to be linearly related to circulating ketone body concentrations, ketone body uptake by skeletal muscle is much lower than that observed by the brain and demonstrates saturation in ketone body uptake between plasma concentrations of ~0.8–1.7 mmol/L [58]. This finding supports earlier work [22] demonstrating that while skeletal muscle is able to extract ~50 % of circulating ketone bodies when concentrations are low (0.1–0.5 mmol/L), this uptake capacity is reduced to only ~5 % when concentrations reach ~6–7 mmol/L [22]. However, exercise may increase ketone body uptake, as recent pilot data [58] and preliminary reports [67] suggest that exercise increases the absolute rate of ketone body uptake and oxidation by skeletal muscle [58, 67]. In addition, ketone body uptake during exercise may also be influenced by the extent of hyperketonemia [33] and how it is induced [45]. For example, when hyperketonemia is induced by short-term starvation, the exercise-induced increase in ketone body uptake is diminished when plasma concentrations exceed ~3–4 mmol/L [33], indicating saturation of ketone body uptake during exercise. However, 2 h of treadmill exercise at 50 % of maximal oxygen uptake (VO_2max_) was shown to stimulate ketone body uptake at the whole-body level when hyperketonemia (~5.5–6 mmol/L) was induced via exogenously provided ketone bodies in subjects studied in the overnight fasted state, but not in subjects who achieved a similar level of hyperketonemia induced by short-term starvation [45]. Another investigation [68] reported that ketone body oxidation rates increased ~three- to fivefold during 30 min of cycling exercise at 60 % VO_2max_ in subjects who had undergone short-term starvation. Specifically, ketone body oxidation rates increased from ~0.44 mmol/min at rest to ~2.1 mmol/min (or ~13 g/h) over the initial stages of exercise, but subsequently decreased to ~1.4 mmol/min (or ~9 g/h). Based on the heat of combustion of the ketone body β-OHB (~19.6 kJ) [16], ketone bodies provided ~85–125 kJ of energy over 30 min of exercise as compared to ~27 kJ during 30 min of rest. Further studies are required to confirm these observations by supplementing ketone bodies during exercise of different duration and intensity to athletes adhering to evidence based fueling strategies.

In addition to differences in ketone body uptake and oxidation rates between various tissues and in response to exercise, studies in rodents have demonstrated that different skeletal muscle fiber types possess different activity levels of the enzymes involved in the utilization of ketone bodies [42, 43]. For example, oxidative muscle fibers have been reported to express higher activity levels of 3-hydroxybutyrate dehydrogenase, 3-ketoacid CoA-transferase, and acetoacetyl-CoA thiolase when compared to glycolytic muscle fibers [43]. In addition, endurance type exercise training has been suggested to increase the capacity of skeletal muscle to oxidize ketone bodies [42, 43, 69–74]. In this respect, endurance type exercise training has been shown to increase the activity of enzymes involved in ketone body metabolism and to attenuate the increase in circulating ketone body concentrations during exercise and post-exercise recovery [42, 43, 72–74]. Therefore, well-trained athletes might be better endowed to utilize ketone bodies as a fuel source [72] and may express a different metabolic response to increased ketone body concentrations compared to that of moderately trained or untrained subjects. However, the few studies that have directly assessed human skeletal muscle [75, 76] or whole body [33, 45, 47, 48, 61] ketone body uptake and/or oxidation rates during exercise have all been performed in untrained subjects.

## Ketone Body Supplements and Exercise Performance

Ketone body supplements have drawn attention as a practical means to induce nutritional ketosis that may have ergogenic effects based on the function of ketone bodies as an alternative and efficient oxidative fuel source, and their capacity to modulate carbohydrate [58], lipid [77], and protein [78, 79] metabolism. Furthermore, as ketone bodies can serve as an alternative fuel for the brain and have been associated with improved cognitive function in patient groups (i.e., Parkinson’s disease, Alzheimer’s disease, epilepsy) [16, 19, 80, 81], ketone bodies have been suggested to enhance performance in endurance athletes via decreases in central (i.e., neural brain) fatigue and improved cognitive functioning [10].

### Ketone Bodies as an Alternative Fuel Source

Ketone bodies have been reported to improve metabolic efficiency (i.e., energetic performance) in animal models [82], primarily through mechanisms involving alternations in glycolytic intermediates and enhanced mitochondrial energetics [16, 82]. Specifically, data obtained from rodents demonstrated a 28 % increase in cardiac efficiency (work in J/mol O_2_ consumed) in response to combined glucose + ketone body (5 mmol/L) administration when compared to glucose alone [82]. The reason for this increase in efficiency has been explained by the higher heat of combustion per C_2_ inherent in β-OHB as compared to carbohydrate derived substrates [16], which is of importance when considering the potential of different mitochondrial substrates on energetic performance. For example, the higher heat of combustion of β-OHB versus pyruvate means that it has the potential to supply more energy to the electron transport chain [16]. As highlighted by Veech [16], pyruvate would liberate ~777 kJ/mole of C_2_ units if combusted in a bomb calorimeter, whereas β-OHB would liberate ~1019 kJ/mole of C_2_ units [16]. Although these findings may provide a rationale to support an ergogenic effect of ketone bodies, there are currently limited human data available on the effects of ketone bodies on exercise metabolism [33, 45, 47, 48, 61] and performance [62] in vivo.

### Influence of Ketone Bodies on Carbohydrate Metabolism

**Fig. 1:**
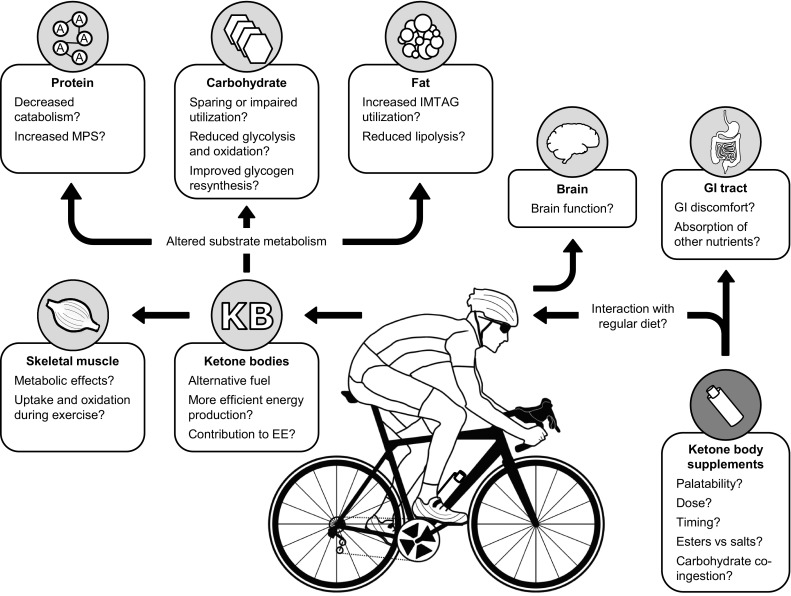
Graphic representation of the potential effects of ketone bodies on exercise metabolism. Important factors for use of ketone body supplements may include taste, dose ingested, timing of intake relative to training/competition, ketone salts versus esters, and co-ingestion with other nutrients (i.e., carbohydrate). These factors may impact gastrointestinal function of the athlete following ingestion. Increased concentrations of ketone bodies during exercise can increase their utilization by tissues such as skeletal muscle and brain. Ketone bodies may also alter the utilization of other endogenous fuel sources including protein, carbohydrate, and fat. *GI* gastrointestinal, *KB* ketone bodies, *EE* energy expenditure, *MPS* muscle protein synthesis, *IMTAG* intramuscular triacylglycerol

The influence of ketone bodies on fuel metabolism during exercise is unclear, in part because much of the available information on ketone body kinetics has been obtained from subjects subjected to prolonged fasting/starvation [33, 47, 48, 61]. Ketone bodies can be readily oxidized as a fuel source by skeletal muscle during exercise [68], and have a similar respiratory quotient to that of glucose (AcAc = 1.0, β-OHB = 0.89) if completely oxidized. By serving as an alternative fuel substrate, ketone bodies may reduce the reliance on glucose utilization and spare endogenous glycogen stores [11]. Alternatively, ketone bodies may compromise endogenous carbohydrate availability via inhibition of hepatic glucose output [58–60] and/or a reduction in pyruvate and lactate oxidation resulting from inhibition of pyruvate dehydrogenase activity [46, 51, 58, 83–86] (for a review see Robinson and Williamson [14]). Therefore, although carbohydrate sparing may benefit endurance performance, it may be hypothesized that ketone body supplementation during exercise reduces carbohydrate oxidation, thereby lowering the capacity to sustain higher intensity efforts [87]. The vast majority of Olympic sports require relatively short-duration, high-intensity efforts. Although these sports are highly dependent on carbohydrate metabolism to sustain high intensity exercise performance, glycogen levels are unlikely to become depleted due to the short duration of competition. Therefore, supplementing ketone bodies as a means to spare carbohydrate utilization in these sports may be irrelevant. The proposed ergogenic effect of ketone bodies when utilized in these sports might be related to the proposed fuel efficiency of ketone bodies. On the other hand, sports such as triathlon and cycling are generally classified as endurance events characterized by high levels of oxidative metabolism in which carbohydrate availability is a major factor limiting performance. For such endurance events, ketone bodies have been proposed to be beneficial to support exercise performance [11]. However, road race cycling also includes repeated sprints and periods of increased workload that exceed an athlete’s maximal aerobic power [88, 89]. These periods of increased exercise intensity often determine the eventual race outcome. Under such conditions, a reduction in glycolytic capacity may compromise an athlete’s specific energy needs during competition. In addition to altering acute exercise metabolism, ketone bodies may augment post-exercise recovery by facilitating the replenishment of muscle glycogen [84]. A recent preliminary report [90] showed that ingestion of a ketone ester beverage resulting in plasma β-OHB concentrations of ~5.3 mmol/L, increased glucose disposal ~33 % and muscle glycogen content ~50 % versus a control beverage after exhaustive exercise [90]. Research is warranted to determine whether the possible benefits of ketone-mediated carbohydrate sparing outweigh the potential adverse effects on the maintenance of high intensity exercise performance (Fig. 1).

### Influence of Ketone Bodies on Fat Metabolism

In addition to altering carbohydrate metabolism, recent data presented in abstract form reported that in comparison to ingestion of carbohydrate, ingestion of combined ketone esters and carbohydrate decreased the respiratory quotient during exercise and decreased intramuscular triacylglycerol (IMTAG) content following 2 h of intense endurance exercise [40]. A greater reliance on endogenous fat sources for energy provision during prolonged exercise could be hypothesized to spare endogenous glycogen stores, thereby improving performance capacity. However, ketone bodies have also been shown to reduce circulatory FFA availability via inhibiting the lipolytic effect of catecholamines [28], and/or via stimulation of hyperinsulinemia, which subsequently reduces lipolysis [25]. Féry and Balasse [45] reported that the intravenous administration of ketone bodies during exercise attenuated the exercise-mediated increase in circulating FFA and glycerol availability, suggesting that ketone bodies may have suppressed the lipolytic effect of exercise [45]. More recently, β-OHB has been demonstrated to inhibit adipocyte lipolysis in vitro via the nicotinic acid receptor protein upregulated in macrophages by interferon-γ (PUMA-G/HM74a) [91]. Collectively, ketone body supplementation may provide an alternative fuel source for working skeletal muscle and alter fuel selection during exercise. However, whether ketone bodies “spare” carbohydrate reserves or impair carbohydrate utilization during higher intensity exercise is unclear. Similarly, whether supplementation with ketone bodies can increase the utilization of IMTAG as fuel or attenuate lipolysis and the availability of FFA during exercise remains to be elucidated (Fig. 1).

## Ketone Bodies in Elite Sports Performance: Focus on Cycling

Although ketone bodies could act as an ergogenic aid for elite endurance athletes whose performance is determined by substrate availability, limited (published) evidence to support this proposed benefit makes advocating the use of such a novel supplement to elite athletes challenging. Supplementation with the recently developed ketone ester drink demonstrates elevated blood ketone body concentrations, with preliminary data indicating altered substrate utilization [40, 92]. When attempting to translate these findings to elite sports performance it is important to consider the practicality of using such a supplement as well as acknowledge any potential negative effects on performance. Timing, dosage, and palatability are important factors to consider when promoting a novel nutritional supplement to elite athletes. Additionally, a comprehensive understanding of the complete metabolic response to ingesting a ketone ester supplement prior to or during endurance performance is essential (Fig. 1). The metabolic demands of endurance sports alone can be highly variable from even paced marathon running and time trial performance to more stochastic intensity observed with biathlon and professional road cycling [88, 89]. Thus, any variation in substrate utilization during performance should be fully understood before attempting to intervene. Current evidence to support the ergogenic effects of a ketone ester supplement is obtained from data reported in a patent application filed in April 2013. The application of a solution containing ~230 kcal from ketone bodies taken prior to exercise in a fasted state subsequently resulted in modest improvements in 30-min rowing performance (averaging ~1 % and up to ~2 %) in 22 elite and sub-elite heavy and lightweight male and female rowers. [62]. However, it is difficult to extrapolate these findings to more prolonged, variable intensity endurance type exercise sports such as cycling during which typical competition duration can range from 1–6 h and where exercise bouts are often repeated over multiple days [89]. Furthermore, when evaluating the ergogenic potential of a nutritional supplement it is important to take into account the day-to-day variability in athletic performance for a given event [93]. Performance improvements in response to a supplement that are within the coefficient of variation observed for a specific sporting event/performance test should be interpreted with caution.

Professional cyclists typically rely on carbohydrate supplements during prolonged multiday stage races such as the Tour de France [65, 94]. In this respect, a reduction in the reliance on carbohydrate metabolism by supplementing ketone esters, may improve performance. For example, retrieving carbohydrate supplements from support staff/vehicles is time consuming, energy inefficient, and often associated with risk of accident or poor positioning during the race. Sparing endogenous carbohydrate stores would, in theory, result in an increased performance capacity during the key parts of the cycling races, e.g. the final hour climb to a summit finish, during which carbohydrate is the dominant substrate. Hypothetically, ketone body oxidation may also permit relatively higher exercise intensity throughout the whole competition in contrast to contributions from fat oxidation. However, until the metabolic interactions of ketone ester supplementation with skeletal muscle fuel selection during exercise are fully elucidated, any proposed ergogenic properties remain theoretical.

## Conclusion

Ketone bodies possess the ability to affect several physiological processes. Previously it has been proposed that ketone bodies can be utilized as an effective energy substrate under certain conditions. As such, ketone bodies have been suggested to have potential positive effects on exercise metabolism and performance. Serving as an alternative fuel source and sparing endogenous carbohydrate stores are among the proposed mechanisms by which ketone bodies have been suggested to benefit endurance exercise performance. Although ketone body supplementation has been proposed to be beneficial for endurance athletes and ketone esters are speculated to be routinely used by professional cyclists, to the best of our knowledge there is currently limited information on the effects of ketone body supplementation on exercise metabolism and performance in recreational and/or elite athletes. Future research should focus on elucidating the metabolic effects of ketone body supplementation during exercise in athletes who adhere to appropriate nutrient intake strategies relevant for their respective sport and/or sports setting. Subsequently, many questions remain to be answered, including practical issues regarding the dose and timing of the proposed ketone (ester) supplement, the interaction with other substrates in various nutritional settings, and their quantitative contribution as an energy substrate during exercise of varying exercise intensity and duration. It will be important to evaluate the kinetics of ketone body availability in a sports-specific manner, tailored towards the needs of the individual athlete. In conclusion, based upon the few available data and our current understanding of ketone body metabolism during exercise in a sports specific setting, we conclude there is currently no evidence to support the use of ketone bodies as an ergogenic aid under conditions where optimal evidence based nutritional strategies are applied.
